# Influence of Cross-Cultural Factors about Sexism, Perception of Severity, Victimization, and Gender Violence in Adolescent Dating Relationships

**DOI:** 10.3390/ijerph191610356

**Published:** 2022-08-19

**Authors:** Isabel Cuadrado-Gordillo, Guadalupe Martín-Mora-Parra

**Affiliations:** Faculty of Education and Psychology, Department of Psychology and Anthropology, University of Extremadura, 06071 Badajoz, Spain

**Keywords:** teen dating violence, gender violence, victimization, hostile sexism, benevolent sexism, culture, mediation effects, moderation effects

## Abstract

The phenomenon of adolescent dating violence directed towards women is a widespread social health problem all over the world. Various investigations over time have analysed and studied this problem from different perspectives, taking into account both the aggressors and the victims. However, apart from these perspectives there are other approaches that have been less explored. This study analyses the phenomenon from a cultural point of view. It looks at the way in which variables such as hostile sexism, benevolent sexism, and the perception of severity attributed to violent behaviour perpetrated by the aggressor can differ depending on the country of origin of a group of victimized adolescent women, specifically, from Spain and Ecuador. The data analysis, based on the construction of a moderated mediation model, revealed that while hostile sexism seems to be linked to a greater extent with traditional cultures, benevolent sexism is highly relevant in today’s society, and modifies the severity that young female victims attribute to violent behaviour and ultimately affects the frequency of victimization. These results revealed the importance of culture and the way in which violence is perceived in different countries as an essential aspect that must be taken into account to guide the construction of effective prevention programs adapted to the specific target groups of adolescents.

## 1. Introduction

In recent decades there has been an exponential growth in research focused on the phenomenon of dating violence. Why adolescents engage in this behaviour, whether as aggressors or victims, and the factors that make these roles persist over time are complex issues to find an explanation for. Given then that any such explanation will not be simple, the interrelation of such variables as the perception of the severity of aggressive behaviour, and sexism are aspects whose exploration could lead to a better understanding of the phenomenon of aggression and victimization.

The first couple relationships are of vital importance in the development of violent behaviour since these experiences become the confirmation or denial of myths existing in society about courting and romantic relationships [1]. Thus, on many occasions, adolescents seem to continue in an abusive relationship with the belief that love can overcome any barrier [2]. This has serious consequences for the victims, since being involved in a relationship where aggressive behaviour is present may decisively modify how they perceive violence and what kind of behaviour is aggressive [3].

The difference between the actual abusive behaviour towards a partner and the couple’s perception and acceptance of that violence has received much attention from researchers. It has been pointed out that the likelihood of exerting some type of aggression against the partner is greater when both members (aggressor and victim) accept this kind of behaviour, so that the victims cannot describe their relationship as abusive [4]. In this sense, being involved in an abusive relationship seems to be related to various factor, such as the presence of violence in their families [5], violence exerted by peers [6], level of dating violence among peers [7], or remaining in an abusive dating relationship for a long period of time which also increases the risk of revictimization [8]. Consequently, these persons are more prone to be involved in this type of relationships, whether as victims or as aggressors [6,9,10]. These facts are aggravated by the difficulty of eradicating the normalization of couple violence, which, according to recent studies, tends to persist for periods of up to six years [11].

Having experienced violence in the past is not the only important variable in explaining tolerance of this behaviour in couples. Another relevant factor is related to sexist attitudes. Arenas-García [12] noted that the degree of assimilation of macho attitudes and beliefs in society plays a fundamental role in the dynamics of couple relationships in adolescence. Through the transmission of sexist attitudinal models, stereotyped functions are attributed to men (strong, active, rational, independent) and women (weak, passive, emotional, dependent) [13], even justifying violence as a way to protect, educate, or dominate the other person based on their gender role within society.

The view of gender roles in dating relationships that is still present in society is directly related to the concept of ambivalent sexism, defined by Glick & Fiske [14]. Those authors pointed out that this particular type of sexism involves an ambivalent, two-component, orientation towards women. The first they called hostile sexism, characterized by the presence of attitudes of domination towards women, based on the premise of male superiority in economic, political, and social institutions. The second they call benevolent sexism, when the man expresses an attitude of protection and affection towards the woman, who depends on the man affectively and sexually. This second type of sexism leads to a devaluation of women, which can be just as negative as that offered by hostile sexism [15].

Studies that have analysed how ambivalent sexism fosters the maintenance of gender stereotypes find that this phenomenon creates a greater acceptance of violence when the said gender prejudices are also present [16,17] In this sense, people who show some type of hostile sexism value non-traditional male and female roles more negatively, while people with benevolent sexism tend to value non-stereotyped female roles more positively [18]. Likewise, hostile sexism encourages the type of male behaviour that gives support to female gender stereotypes, rewarding behaviour in women such as raising children, doing household chores, or caring for or being available to men [19]. These types of behaviour, however, limit women’s freedom, thus constraining their role in society.

On the other hand, benevolent sexism encourages the idea in some women that they should be protected by their partner [20], thereby creating a link between hostile and benevolent sexism, even though they might in principle seem radically opposed. Thus, authors such as Sibley, et al. [21] state that high levels of benevolent sexism in women predict increases in the levels of hostile sexism. Similarly, studies such as the one conducted by Sibley & Becker [22] in Australia find that few people endorse only one type of sexism.

The idealization that some adolescents and young adults have of couple relationships should be added to these sexist stereotypes, especially in young women [23]. This idealization justifies violent behaviour based on the myths of “romantic love”, and behaviours such as jealousy, psychological control, or emotional blackmail are not only endorsed but even desired [24,25,26]. With this, violence seems to be justified to the extent that it coincides with what is established by social norms [27]. The result of these facts is the normalization of violence as a normal way of interacting within the couple, making the abuses invisible [28].

Nonetheless, how adolescents perceive violence and their acceptance of acts of aggression, or the gender roles and sexism present in society, are not the only factors to consider. Contextual and cultural variables might also have an important role that ought to be taken into account in the development of this phenomenon. In this sense, Archer [29] showed how the levels of gender equality in a country, or the presence of a higher level of individuality in a culture, are related with less victimization of women. Therefore, finding the inequalities that the culture can cause becomes an important factor to explore, not only for the comprehension of the phenomenon, but also for the establishment of adequate measures of prevention and intervention.

Spain is a traditionally conservative country that to a certain extent has maintained the gender roles associated with the masculine and the feminine [30]. However, research such as that carried out by Martínez, et al. [31] has noted some evolution away from what was the case in the past. Hence, adolescents tend to be less represented than adults by the image of the characteristics associated with masculine and feminine roles. Similarly, it must be added that gender gap in Western European countries (such as Spain) is one of the lowest in the world. Specifically, the Global Gender Gap Report of 2017 [32] records a gender gap of 25%, being consequently placed ahead of North America. Nevertheless, with respect to dating relationships, the survey on Perception of Gender Violence in Adolescence and Youth of the Spanish Government Delegation for Gender Violence [33] indicates that one in every three adolescents and young people accept that there may be some kind of control in the couple, especially regarding the things that can or cannot be said or the time to leave and to get back home. Likewise, subsequent studies, such as the one carried out by the Scopio Project of the Centro Reina Sofía in Madrid [34], show how, despite the fact that 87% of the young people surveyed consider gender violence to be a very serious social problem, 27.4% of them believe that it is normal in the couple, and 21.2% consider that it is a politicized issue which has been exaggerated.

On the other hand, Ecuador is an even more conservative country regarding gender roles and sexist attitudes. In this sense, its social and cultural characteristics are of vital importance for the development and maintenance of these attitudes, as has been noted by authors such as [35,36,37], who explain the importance of sexist attitudes in the role played by women in Ecuadorian culture and in domestic violence. In this sense, recent studies such as the one carried out in Quito (Chile) by [38], found a tendency in adolescents which revealed how this group of people accept in general the traditional gender role model. Thus, the Global Gender Gap Report of 2017 [32] records a gender gap in Latin America countries of 29.8%, a higher gap than Spain. Likewise, Guarderas [39] comments that the efforts carried out by the government of Ecuador in favour of human rights and security when addressing gender violence in the criminal sense have not achieved any profound modification of the patriarchal conceptions. Rather, those efforts have tended to maintain the status quo. These findings are supported by [40] who comment on the results of the 2011 Survey on Family Relations and Gender Violence carried out with 70,446 Ecuadorian women over the age of 15, with 60% having suffered gender violence. The figures were higher for those women who were divorced (85.4%) or separated (78%). Although there were no data for adolescent couples, it was found that women who got married for the first time between the ages of 16 and 20 were those who suffered the most violence (70.5%). This datum is important since 25.8% of Ecuadorian women who marry, do so when they are in that age group.

Despite all the evidence, not enough is known about the factors that contribute to victimization and tolerance of dating violence during adolescence [41,42]. Although it is true that sexism clearly seems to play a significant role in the aggressor’s perpetration of violent behaviour [43,44] and in the victim’s acceptance of it [45,46], there remain many aspects to explore about how these variables interrelate to conjointly influence the lack of recognition of abuse.

### The Study

In this sense, it is important to consider how the specific cultural characteristics associated with different countries can cause differences between the establishment of the phenomenon of women victimization within the couple. In this regard, for example, the recent study carried out by [47], indicates how the level of sexism in the Spanish population and immigrant population is different, with immigrants having a greater tendency to show beliefs and attitudes of tolerance towards violence directed to women. This fact could be explained considering the results of studies which have pointed out how cultural patterns make it possible for a society to function as it provides its members with behavioral guidelines [48]. These cultural patterns include guidelines about behaviors such as the way people interact with each other. This way, sexism is recognized as a cultural pattern which promotes violence to women [49]. This tendency is expected to be even more prevalent when comparing teenage dating violence in different countries with different cultural guidelines which, we expect, will modify, the relationships established among all the variables include in this study As a consequence, understanding the interconnections between sexism (both hostile sexism and benevolent sexism), the severity attributed to abusive behavior in dating, nationality and how these variables interact among each other would be of great value, especially from the point of view of the female victims.

The objective of this present study was to contrast in a sample of both Spanish and Ecuadorian adolescents, with different cultural background, to what extent in their dating relationships their nationality as well as the level of hostile and benevolent sexism showed by women victims act as mediatory and modulatory factor modifying the victimization suffered. The following hypotheses were posited:

**H1:**  *Perception of severity will have a direct influence in the frequency of Victimization.*

**H2:**  *Hostile and Benevolent sexism will be mediatory factors in the relationship between the Perception of severity perceived in violent behaviour and the frequency of Victimization indicated by victims.*

**H3:**  *Nationality of victims will modulate the relationship between the Perception of severity of violent behaviour and the frequency of Victimization, modifying the mediatory effects caused by hostile sexism and benevolent sexism*.

## 2. Materials and Methods

### 2.1. Sample

The original sample of the study consisted of a total of 1286 male and female adolescents from Spain and Ecuador aged 14–21 years (M = 17; SD = 2.1). Considering the objectives of the study, all male adolescents were eliminated to study the characteristics of intimate partner violence against adolescent women and how the different variables considered interact. As a result, the final sample of this study consisted of a total of 687 female adolescent participants aged 14–21 years (M = 17.2; SD = 2.1). The Ecuadorian participants, 350 in total had a mean age of 16.5 years (SD = 1.4). The Spanish participants, 337 in total, had a mean age of 17.8 (SD = 2.5).

The selection of the sample included an approximately proportional stratified procedure that included different Spanish and Ecuadorians secondary schools. In Ecuador, two schools were selected from the Metropolitan District of Quito, one from a rural area, and one from an urban one. In Spain, three schools (one rural, two urban) were selected from the Autonomous Community of Extremadura (Spain). In the urban areas, schools were selected in both the city centre and its periphery with the objective of covering diverse socioeconomic contexts. The choice of both rural and urban areas also had the objective of including populations with different incomes. In rural areas, the family income level was below the average for the region, and approximately half of the parents of the participants did not have a university education. In the urban areas, the schools were selected from both residential zones where the purchasing power was medium-high, and humbler zones where families worked in unskilled jobs with medium to low incomes. The questionnaires were administered from the second year of secondary school (14 years old). On the other hand, the universities have students from medium and medium-high socio-economic groups, whose parents have stable jobs. However, there are also students with low incomes. The questionnaires at university were administered in the first course.

### 2.2. Instruments

Two questionnaires were administered:

Dating Violence Questionnaire, CUVINO [50]. This questionnaire comprises 61 items, grouped into three thematic blocks. In this study we only used the first block. The first of these blocks is subdivided into two: one is designed to measure the frequency of the violence, and the other, the perception of severity that this behavior provokes or might provoke in adolescent. In both cases, a 5-point Likert scale is used, but with different anchors—in the first case (frequency of victimization) from “Never” to “Almost always”, and in the second (perception of severity) from “None” to “A lot”. The modalities of aggression considered in this first thematic block are eight for both frequency and perception of severity: detachment (“Is a good student, but is always late at meetings, does not recognise his/her promises, and is irresponsible”), humiliation (“Ridicules your way of expressing yourself”), sexual (“You feel forced to perform certain sexual acts”), coercion (Threatens to commit suicide or hurt himself/herself if you leave him/her”), physical (“Has thrown blunt instruments at you”), gender (“Has ridiculed or insulted women or men as a group”), emotional punishment (“Refuses to give you support or affection as punishment”), and instrumental punishment (“Has stolen from you”).

The second thematic block focuses on the perception that adolescents have of themselves as abuse victims. And the third thematic block delves further into the abusive relationship, addressing different aspects (duration of the relationship, attempts to break up, etc.).

There are two validation studies of CUVINO in Spanish. The first version of CUVINO [51] included 62 behavioural indicators. On the other hand, the second [50], which was validated in Argentina, Mexico and Spain, reduces the number of items to 42 (this second version was the one used in the present study).

In both versions, an exploratory factor analysis showed a structure of eight factors: detachment, humiliation, sexual violence, coercion, physical and gender violence, emotional and instrumental punishment. The fit of the questionnaire to the present study samples (Spanish and Ecuadorian) is evidenced in the reliability indices (Cronbach’s α) obtained, which exceeded 0.92 in both cases. Considering the reliability index presented by each of the factors that made up the questionnaire, we found Cronbach’s α for the sample from Ecuador to range from 0.59 in the ‘instrumental’ factor to 0.82 in the ‘humiliation’ factor. In the case of the Spanish sample, the indices were from 0.64 in ‘instrumental’ abuse to 0.90 in gender violence.

Scale for the Detection of Sexism in Adolescents [52]. This scale assesses the sexist attitudes that adolescent have towards traditional gender traits and roles, considering two sub-scales: hostile sexism and benevolent sexism. The hostile scale refers to those traditional stereotypes which place women in an inferior position. For example, one item which measures hostile sexism is “The best place for women is their house and their families”. The benevolent sub-scale focusses on the beliefs which highlight the qualities of women supporting the raising of children and care for the family, needing protection from men. For example, an item of the scale which measures benevolent sexism is “Women are weaker than men in every aspect”. The scale used a six-point Liker type, with 1 being “totally disagree”, and 6 being “totally agree”.

For the reliability of the scale, the Cronbach’s α values were 0.89 for the total scale in both samples, 0.80 and 0.83 (Ecuador and Spain, respectively) for the hostile sexism and 0.91 and 0.88 (Ecuador and Spain, respectively) for the benevolent sexism factors. Its structure showed conformity with Ambivalent Sexism Theory [14], and convergent validity with the Ambivalent Sexism Inventory (ASI) adapted and validated in Spain by [53].

### 2.3. Procedure

Before starting with the distribution and administration of the questionnaires, the research objectives, procedure, instruments, and techniques to be used were verified and approved by the Bioethics Committee of the University of Extremadura (Spain). Consent of the Educational Administration was obtained to go to the schools in two differentiated phases. The first included a detailed report about the objectives and methods of the research that was sent to the inspection services of the Metropolitan District of Quito and the Regional Government of Extremadura, together with the ethical principles guiding the research. The approval of this report allowed access to the schools. In the second, the conformity of the schools’ management teams was requested to facilitate access to the classes during school hours.

Once the consent of the Educational Administration had been obtained, the parents were asked to give their permission (due to the participation of minors), as also was the Regional Educational Administrations (inspectors and teachers). The parents were sent a letter describing the study and the mechanisms used to guarantee the anonymity and confidentiality of the pupils’ responses. Additionally, they were also informed that the distribution, collection, storage, and analysis of the responses would only be carried out by the researchers responsible for the project, and that no other person would read or know the responses that the pupils gave to the questionnaire. The letter was accompanied by an authorization form for the parents to sign and return if they wanted their children to participate.

Once all the authorizations had been obtained, the questionnaire was distributed on paper to the pupils who participated voluntarily, with their anonymity guaranteed. The time required for them to fill out the questionnaires was around 50 min.

One researcher at least remained in the classroom while the pupils completed the questionnaires in order to resolve any doubts that might arise during the process. The classrooms where the questionnaires were completed are regular classrooms. This way, all the students had their own desk and chair (both male and female), so that the questionnaires were completed individually and in silence. Finally, the instructions provide by the researchers were the same for all the adolescents.

### 2.4. Data Analysis

The preliminary analyses were carried out beginning with the identification of the victimized women, and their classification into two categories. Adolescent women who scored 0 on all the CUVINO scales were not considered to be victims of gender violence. Those who had scores greater than 0 were assigned to two different groups. Those with scores of less than 3 formed the “Sometimes” victimization group, and those with scores greater than 3 were classified as “Frequent” victims.

Secondly, descriptive and correlation analyses were carried out using the mean and standard deviation, as well as the Spearman correlation coefficient for each of the study factors intended to be put into the theoretical model.

Likewise, to check for the existence of an association between victimization (“Sometimes” and “Frequent”) and nationality (Spain and Ecuador), the chi-squared test was performed, contrasting the measure of the association using Cramer’s V coefficient. These analyses were performed with the statistical package IBM SPSS Statistics 22 (IBM Corp., Armonk, NY, USA, 2012).

**Figure 1:**
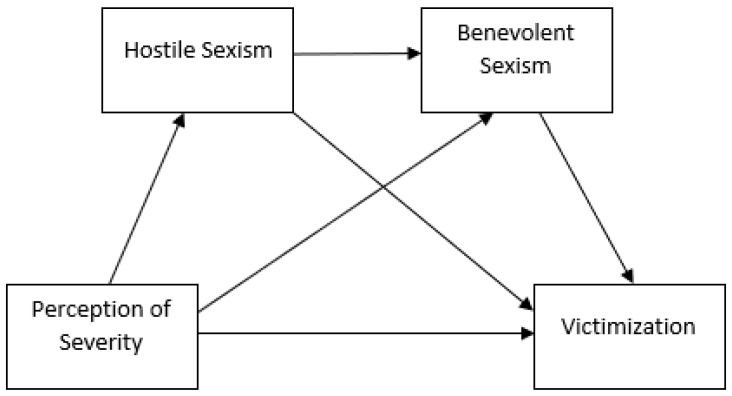
Serial mediation model 6.

Finally, a serial mediation model was constructed using “Process” v3.3 [54] to assess the mediating effect of hostile sexism (first mediator) and benevolent sexism (second mediator) and Victimization (independent variable) (Figure 1). This model predicts victimization through the perception of severity, with this variable being mediated by hostile sexism and benevolent sexism, as well as by the interaction between the two. For this, the theoretical model (Model 6) shown in Figure 1 was followed.

**Figure 2:**
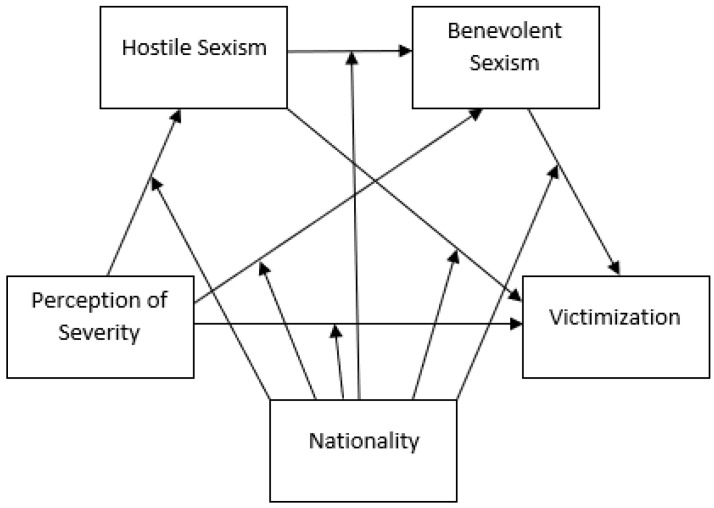
Moderate mediation model 92.

Finally, the moderation effects were analysed with Model 92, in order to analyse whether nationality influenced the associations of the variables in the study (Figure 2). This model predicts victimization through the perception of severity, considering the mediation effects of hostile sexism and benevolent sexism, with these relationships being moderated by nationality, as well as the interaction among all the variables.

“Process” is a macro for use in SPSS that uses least squares regression to estimate the importance and size of direct and indirect effects in mediation models. “Process” performs better than a traditional causal step approach (both in terms of statistical power and type I error). The indirect effects are inferred using the bootstrapping method after generating an empirical representation of the sampling distribution of the indirect effects. Bootstrapping is suitable for linear hypotheses when the variables are not normally distributed [55], as was the case in the present study. All the variables were standardized before the analysis to facilitate the interpretations of the results.

## 3. Results

The analysis of the results revealed that 426 of the total of 687 adolescent women who made up the study sample had suffered violence in a dating relationship, with detachment, coercion, and emotional punishment being the types of violence that they suffered most.

Focusing on the victims, Table 1 presents the data of the frequency of victimization. In this sense, it was found that Spanish and Ecuadorian girls present a similar level of victimization when the violence suffered is “Sometimes” (frequency of violence less than 3). In contrast, it was observed that a greater number of Ecuadorian adolescents suffered “Frequent” violence (frequency of violence greater than 3).

Likewise, the analysis of the relationship between variables using the χ² statistic reveals a statistically significant relationship between the nationality of origin (Spain and Ecuador) and the degree of victimization (Sometimes and Frequent) (χ² = 8.077; *p* < 0.01). The strength of the association measured using Cramer’s V coefficient is moderate (V = 0.238; *p* < 0.01).

The descriptive analyses of the variables in the study are presented in Table 2. Ecuadorian girls showed higher degrees of hostile sexism (SH) and benevolent sexism (SB). Likewise, the data analysis revealed that the perception of severity with respect to violent behaviour was greater in the Spanish adolescents.

The analysis of the correlation matrix (Table 3) indicated that SH shows a positive relationship with victimization. Similarly, SB shows a statistically significant and positive relationship with victimization. With this, the adolescents who present a greater degree of SH and SB score higher in victimization. Finally, it was found that the perception of severity that violent behaviour provokes in the adolescents correlated negatively with all the variables in the study. This fact indicates that adolescents with higher perception of severity scores are those presenting lesser victimization, SH, and SB.

A serial mediation analysis was performed with 10,000 bootstrap samples. The mediation model coefficients are provided in Table 4. The direct effect of the perception of severity was significant (*β* = −0.11; t = 5.22; <0.01).

Subsequently, the models of the mediating variables, SH and SB, and the dependent variable, annoyance of violence, were analysed.

Hostile sexism was significantly associated with the degree of victimization and the perception of severity caused by violence. Benevolent sexism was significantly associated with victimization and perception of severity. After controlling for the effects of mediators, the direct effect of perception of severity on victimization was still significant.

To evaluate the indirect effect and the confidence intervals (CI), a bootstrap procedure was used. An indirect effect is significant if the CI does not include the value 0. For the pathway “perception of severity → hostile sexism → victimization”, a significant indirect effect *β* = −0.043 was obtained; 95% CI [−0.0727, −0.0194].

For “perception of severity → benevolent sexism → victimization”, a significant indirect effect *β* = −0.012 was obtained; 95% CI [−0.0259, −0.0004].

Finally, for “perception of severity → hostile sexism → benevolent sexism → victimization”, a significant indirect effect *β* = −0.026 was obtained; 95% CI [−0.0389, −0.0137]. Therefore, SH and SB have a partial mediating effect on victimization.

The moderation effects indicate that nationality was found to be a determinant variable in the effects of SH and SB on victimization. The coefficients of the moderated mediation model are given in Table 5.

In this way, it can be observed that nationality has a statistically significant influence on SH (*β* = 1.007; t = 4.40, *p* < 0.001). Nonetheless, the indirect effects show how the “perception of severity → hostile sexism → victimization” pathway was only significant in the case of Ecuadorian adolescents (*β* = −0.05; 95% CI [−0.0881, −0.0255]), but not in the case of Spanish adolescents (*β* = −0.01; 95% [−0.0403, 0.0051]).

It was found that nationality does not have any statistically significant effect on SB (*p* > 0.05).

Finally, the analysis of the results revealed that nationality has a statistically significant influence on victimization (*β* = −0.52; t = −2.11; *p* < 0.05). The indirect effects show how the “perception of severity e → hostile sexism → benevolent sexism → victimization” pathway is significant both for Spanish (*β* = −0.015; 95% CI [−0.0345, −0.0031]) and for Ecuadorian adolescents (*β* = −0.016, 95% CI [−0.0286, −0.0072]).

## 4. Discussion

Authors should discuss the results and how they can be interpreted from the perspective of previous studies and of the working hypotheses. The findings and their implications should be discussed in the broadest context possible. Future research directions may also be highlighted.

The complex network of relationships that affects the process of victimization in adolescent dating has revealed how variables such as sexism (both hostile and benevolent), perception of severity and nationality are factors that are important to consider in the prediction of victimization. It was found that these variables interact with each other, some also becoming moderating and modulating variables that modify other variables’ direct effect on victimization.

The first hypothesis, which refers to the perception of severity of violent behaviour being a factor that influences directly the frequency of victimization is confirmed, as those who perceived less severity in the abusive behaviour perpetrated in couples were more frequently victims of violence. Thus, how the female adolescents interpret the gravity of violent behaviour perpetrated within a dating relationship does influence their taking on the role of victim. As a result, having a high tolerance of violence could be the key that explains why many victims are not able to recognize themselves as victims. In this way, some authors have revealed that between 70% and 80% of women victims of abuse did not recognize the violence used against them [56]. Subsequently, Rodríguez-Franco et al. [57] highlighted the inconsistency between objective indicators of victimization and the self-perception of violence in both adolescent and young adult couples, with only 6% of victims perceiving themselves as such compared with the 41–77% of victims who have no perception of any abuse. This fact could also be related to the type of violence suffered more frequently. In this way, the results indicate that the most common types of abuse involve behaviors of psychological violence such as detachment, coercion, and emotional punishment, with physical aggression being rare. This kind of aggression, which does not leave a visible mark, seems to be interpreted by female adolescent as normal and insignificant behavior within a romantic relationship, going unnoticed by the victimized adolescents themselves [58], thereby promoting a lesser perception of severity.

This fact has obvious implications in the phenomenon because, victims who do not identify themselves as such, cannot ask for help. In this sense, Koepke, et al. [59] pointed out that the aggressor’s own sexist attitudes may be what provokes the victim’s approval, who ends up normalizing their partner’s behaviour and attitudes, thereby perpetuating their own role as victim without realizing it [60].

With respect to the second study hypothesis, which refers to sexism as a mediatory factor in the relationship between “Perception of severity” and “Victimization”, has been also confirmed. In particular, benevolent sexism and hostile sexism interact with perception of severity, altering the way in which these variables contribute to the prediction of victimization.

As was expected, ambivalent sexism is an important variable that affects the victimization process in dating relationships, thus confirming previous research [30,61] about the importance of sexism and its relationship with the presence of higher rates of violence. In this sense, the results of the present study point in the same direction as those of researchers such as [62], or [47] who indicated that sexism, although not a causal element, when combined with other factors increases the likelihood of the use of violence in couples.

Starting with hostile sexism, the relationship found between this variable, perception of severity and victimization could be related with the way violence is perceived within the couple. In fact, it has been pointed out that people who assume stereotyped roles (that is, those related with hostile sexism) are therefore more tolerant of violence within couples and have a greater acceptance of the use of aggression, and of a woman being abused than of a man, whether psychologically, physically, or sexually [63]. Female victims could have assumed this belief as their own, especially in traditional cultures such as Latin societies. This conclusion has been already pointed out by authors such as [38], who indicated that adolescents tend to assume the traditional gender role model. In this regard, Fernández et al. [64] pointed out that, although girls in general showed a lower degree of acceptance of abusive behaviour and a higher level of perception of severity, as the frequency of abuse increased, the victimized girls also began to show greater acceptance of violence perpetrated by their partners and more sexist attitudes, which leads to a progressive normalization of violent behaviour. Additionally, the importance of sexism and sexist attitudes as a mediatory variable in the recognition of abuse may be related to the greater difficulty that female victims have in labeling certain types of behaviour as sexist when the perpetrator is their partner or someone they are attracted to [65,66].

On the other hand, the fact that victims attribute less severity to the abusive behavior could be related to the idealization of love and relationships, as well as the presence of benevolent sexism [4,23,67] found both in Spain and Ecuador, facts that may be even more pronounced during adolescence due to the inexperience of persons in these age ranges. In turn, this idealization is linked to gender stereotypes that characterize men and women differently.

Benevolent sexism becomes a mediatory factor for both Spanish and Ecuadorians victims. In general, it is common to find that these subtle sexist attitudes are related to greater satisfaction in romantic relationships [68,69]. With this, it has been additionally pointed out that women would not only present benevolent sexism themselves, but would also feel more attracted to those men who support these principles [70]. When this behaviour is common in a person’s life, it is difficult for them to detect it, and it would be equally difficult for them to perceive the severity of its negative consequences [71]. Similar results were suggested by [64], who indicated that increased frequency of violence implies a greater acceptance of abuse, all of which is reinforced by the presence of sexist attitudes in the victims who hide the situation of abuse they are living with because they consider such abuse to be normal behaviour. The link between sexism and romantic love highlighted by such authors as [72,73] could be the key to understanding this phenomenon, even more so considering that it is the girls who show greater idealization of love, while boys tend to adopt a paternalistic vision of care and dependence with respect to their partner [74]. With this, sexist attitudes could activate certain mechanisms in a victim that stop them from labeling themself as abused. Consequently, seeking help or reporting the abuse suffered would be unlikely due to the victim’s lack of recognition of the problem. These facts consequently affect the frequency of victimization and the persistence of the victim role.

In view of the above, the finding linked to the relationship among perception of severity, hostile sexism, benevolent sexism, and victimization for both, Spanish and Ecuadorians female victims, seems to go in the same direction of previous results explained. Actually, it has been pointed out that the acceptance of violence (which cause less perception of severity), which is related with the assumptions of gender roles and myths about romantic love (both linked with hostile and benevolent sexism stereotypes), contributes to justifying or minimizing the consequence of the suffering, making the gender violence an invisible type of abuse [16,28].

Finally, nationality seems to take an important part as a modulatory factor in the web of relationships created, confirming partially the third hypothesis. Consequently, being a Spanish or an Ecuadorian victim modifies the way the hostile sexism changes the relationship between the perception of severity and victimization. The combination of hostile sexism and a weaker perception of severity establishes an especially relevant link in more traditional cultures and societies in which gender roles are seen as normal aspects associated with what is masculine and what is feminine. In this way, the fact that behaviours like controlling mobile phone access are classified as a manifestation of love by adolescents in Latin American countries such as Bolivia, Colombia, Cuba, El Salvador, and Nicaragua, among others, is an example of how these young people tend to reproduce the social norms that even today continue to feed violence against women [75]. Ecuador, being part of Latin American context, also has a traditional vision of gender roles, and reproduces similar cultural patterns. In this regard, authors such as [76] came to connect these facts with widely extended cultural factors such as the so called “marianismo”, a current of thought present in areas of Latin America which generates sexism. Regarding this, authors such as [42] pointed out that religion in Latin American countries is related to a greater justification of violence towards women within the relationship. In this sense, the importance of this situation has even been reflected in how, even though 18 Latin American countries have modified their laws in an attempt to tackle the problem, their results to date have not been fruitful [77].

These findings point to an essential difference with those found in Spain where, although it is true that gender stereotypes are still present, the evolution of masculine and feminine characteristics has led to the generation of fewer stereotypes, at least in the vision that adolescents have of themselves [32]. Furthermore, most adolescents know what gender violence is, and can recognize it, but, at the same time, they consider this phenomenon a problem linked to older people [78]. As a result, the more recognizable forms of gender stereotypes related with hostile sexism could have less importance in countries such as Spain. In contrast, the perpetuation of the assumption of traditional gender roles, which seems to be accepted by society in general, and adolescents in particular in Ecuador [38], may be the key to the maintenance of both gender gap and traditional gender stereotypes.

Therefore, although women tend to be discriminated in different levels (legally, in the labor market, etc.) in society [79], hostile sexism may be restricted to traditional countries. Meanwhile, benevolent sexism, a more hidden way of assuming the patriarchal beliefs, seems to lead to a significant increase of gender violence among adolescents [80] in non-traditional cultures as well as in the traditional ones. The belief that equality for women has already been achieved and that, therefore, no further political measures are needed for this purpose, and would increase the difficulties to really achieve equality [81,82], causes violence directed towards women to continue to perpetuate itself in society.

These facts are confirmed in the present study which pointed out the presence of benevolent sexism in the sample of adolescent from both countries, in the same way that previous studies carried out by different authors [64,67,83,84]. Consequently, benevolent sexism shows its importance in today’s actual society, connecting additionally with the romantic myths about love and the necessity to stablish intimate connections [85].

## 5. Limitations

The study has some limitations which should be considered. First, its transversality. A longitudinal study could provide more data on violence of these adolescents as adults and whether the prevention measures help to avoid the escalation of abuses in their future romantic relationships. Also, having the perspective of aggressors, victims-aggressors, and male victims could represent a line of work to analyse. Additionally, having a deeper knowledge about the way young people use social networks is another key aspect in today’s society. Analysing the risks involved in the misuse of these technologies in the adolescent population (especially pornography) is a relevant line of future research that could guide the construction of affective-sexual education programs contextualized in the current technological world. These limitations should guide future studies to obtain complementary results to those showed in the present study to obtain deeper analysis of the phenomenon of teen dating violence, victimization, and aggression.

## 6. Conclusions

The association between sexism and dating violence has been extensively studied. Nonetheless, dating violence in adolescent couples has to date been a less explored field, especially from the victims’ point of view. One of the major contributions of this study is its focus on the victims in an attempt to understand which variables become intertwined to form causes contributing to abuse. Additionally, another major contribution is linked to the fact that the study seta a good example of how important cross-cultural comparisons are to understand the different gender approaches in diverse cultures, which determine different prevention and intervention protocols. Likewise, the links between variables, and how some of them can influence others, show the importance of looking for complex explanations instead of focusing on simple, one-way, relationships. Sexism, as a mediatory factor of the perceived severity of this type of behaviour, is a key factor to understanding this phenomenon. However, while hostile sexism shows its influence in traditional cultures, the role of benevolent sexism is important in today’s actual society, especially during teenage years in which people are developing and tend to assume the values of society. This way, although the prevalence of benevolent sexism seems to be higher in traditional cultures (since hostile sexism and benevolent sexism correlate with each other), in non-traditional cultures benevolent sexism is present as well and shows its influence in teen dating violence and on how female victims accept the abuses as part of the relationship. This fact suggests the need to modify the way in which gender roles, equality and the needs of women are analysed in order to truly achieve a comprehensive and multidisciplinary understanding of the problem. These findings can help guide actions of both prevention and intervention.

Consequently, understanding the cultural differences among countries becomes a key factor in designing prevention and intervention programmes. Likewise, it seems to be key elements in the approach to solving the problem of violence in adolescent dating and its serious consequences could be to implement prevention programs focused specifically on sexism, its deconstruction, and its consequences, and to promote coeducation models from early childhood onwards, and work in schools of topics such as empathy, equality and respect in both formal and informal contexts. However, these prevention programs should be contextualized considering the culture and sub-cultures where they are inserted, not it should not be assumed that the characteristics of adolescents are similar just because of their age.

## Figures and Tables

**Table 1:** Sociodemographic data of the sample.

	Studies		Family Income	
Spain	Secondary School	37%	−900 €	20.3%
	Baccalaureate	43%	900–2500 €	63.5%
	University	20%	+2500 €	13.5%
Ecuador	Secondary School	61%	−900 €	55%
	Baccalaureate	25%	900–2500 €	36%
	University	14%	+2500 €	9%

**Table 2:** Frequency of violence.

		Sometimes	Frequent	Total
Nationality	Spain	199	4	203
		49.3%	18.2%	47.7%
	Ecuador	205	18	223
		50.7%	81.8%	52.3%
Total		404	22	426
		100.0%	100.0%	100.0%

**Table 3:** Descriptive Statistics.

			Spain		Ecuador		Correlations		
	Minimun	Maximun	X	SD	X	SD	1	2	3
1. V	1	5	1.30	0.50	1.58	0.68	-		
2. SH	1	6	1.50	0.77	2.44	0.86	0.56 **	-	
3. SB	1	6	2.51	1.15	3.88	1.12	0.51 **	0.68 **	-
4. PS	1	5	3.93	1.19	3.05	1.38	−0.41 **	−0.44 **	−0.36 **

V = Victimization; SH = Hostile Sexism; SB = Benevolent Sexism; PS = Perception of Severity; ** *p* < 0.01.

**Table 4:** Mediation model.

	Hostile Sexism		Benevolent Sexism		Victimization	
	*β*	t	*β*	t	*β*	T
Perception of Severity	−0.28 ***	−9.16	−0.09 *	−2.38	−0.11 ***	−5.22
Hostile Sexism			0.77 ***	13.05	0.15 ***	4.43
Benevolent Sexism					0.12 ***	4.92
R^2^	0.16		0.36		0.31	
F	83.92		120.59		65.55	

* *p* < 0.05; *** *p* < 0.001.

**Table 5:** Moderated mediation model.

	Hostile Sexism		Benevolent Sexism		Victimization	
	*β*	t	*β*	t	*β*	t
Perception of Severity	−0.08	−0.84	−0.26 *	−2.38	−0.14 *	−2.13
Nationality	1.01 ***	4.40	0.50	13.05	−0.52 *	−2.11
Interaction Perception of Severity × Nationality	−0.07	−1.13	0.13	1.59	0.02	0.56
Hostile Sexism			0.77 ***	3.75	−0.09	−0.81
Benevolent Sexism					0.17 *	2.09
Interaction Hostile Sexism × Nationality			−0.11	−0.84	0.17 *	2.41
Interaction Benevolent Sexism × Nationality					−0.02	−0.33
R^2^	0.31		0.43		0.34	
F	64.42		63.53		30.86	

* *p* < 0.05; *** *p* < 0.001.
